# Outbreaks of virulent diarrheagenic *Escherichia coli *- are we in control?

**DOI:** 10.1186/1741-7015-10-11

**Published:** 2012-02-02

**Authors:** Dirk Werber, Gérard Krause, Christina Frank, Angelika Fruth, Antje Flieger, Martin Mielke, Lars Schaade, Klaus Stark

**Affiliations:** 1 Department of Infectious Disease Epidemiology, Robert Koch Institute, DGZ-Ring 1, 13086 Berlin, Germany; 2 Department of Infectious Diseases, Robert Koch Institute, Nordufer 20, 13353 Berlin, Germany; 3 National Reference Center for Salmonella and other Bacterial Enteric Pathogens, Robert Koch Institute, Burgstrasse 37, 38855 Wernigerode, Germany; 4 Robert Koch Institute, Nordufer 20, 13353 Berlin, Germany

**Keywords:** epidemiology, public health, E. coli, Escherichia coli O157, disease outbreaks

## Abstract

Shiga toxin-producing *Escherichia coli *(STEC) are the most virulent diarrheagenic *E. coli *known to date. They can be spread with alarming ease via food as exemplified by a large sprout-borne outbreak of STEC O104:H4 in 2011 that was centered in northern Germany and affected several countries. Effective control of such outbreaks is an important public health task and necessitates early outbreak detection, fast identification of the outbreak vehicle and immediate removal of the suspected food from the market, flanked by consumer advice and measures to prevent secondary spread.

In our view, opportunities to improve control of STEC outbreaks lie in early clinical suspicion for STEC infection, timely diagnosis of all STEC at the serotype-level and integrating molecular subtyping information into surveillance systems. Furthermore, conducting analytical studies that supplement patients' imperfect food history recall and performing, as an investigative element, product tracebacks, are pivotal but underutilized tools for successful epidemiologic identification of the suspected vehicle in foodborne outbreaks. As a corollary, these tools are amenable to tailor microbiological testing of suspected food.

Please see related article: http://www.biomedcentral.com/1741-7015/10/12

## Introduction

Among diarrheagenic *Escherichia coli*, those producing Shiga toxin (synonym: Vero toxin), are the most virulent to date. These Shiga toxin-producing *E. coli *(STEC) can cause hemorrhagic colitis that may manifest as painful, grossly bloody diarrhea [1] as well as hemolytic uremic syndrome (HUS) - a potentially fatal thrombotic microangiopathy, typically affecting children (pathogenesis and treatment strategies are fully discussed in the accompanying commentary by Goldwater *et al. *[2]). The case-fatality ratio of STEC illness is dependent on the patients' age and the virulence profile of the infecting strain. It is less than 1% for STEC gastroenteritis [3]. For apparently sporadic STEC-associated HUS, the case-fatality ratio in the acute phase is between 2% and 5% [3,4], but it can be as high as 10% in outbreaks of the rare sorbitol-fermenting O157:H- STEC [5].

Since their first description in 1977 [6], many (> 100) different STEC serotypes, a categorization based on O (somatic) and H (flagellar) antigens, have been associated with human disease. Of those, O157:H7 has the strongest association with HUS worldwide [7]. This serotype is the primary target for diagnosing human STEC infection in many countries due to its virulence and propensity to cause common source outbreaks coupled with its ease of diagnosis by culture isolation. In many countries, infection with STEC O157, regardless of the H-antigen, is probably less frequent than infection with STEC of other serogroups. These 'non-O157 STEC' constitute a heterogeneous group of organisms, which have, on the whole, a lesser risk of causing bloody diarrhea [8,9] and HUS [9]. For example, prior to the large STEC O104:H4 outbreak in 2011 (see below), non-O157 STEC accounted for more than 80% of reported STEC infection, but only for approximately 1/3 of STEC-associated HUS in Germany [4,10].

Stools submitted for diagnosis of acute community-acquired diarrhea, even when bloody, are not always investigated for the presence of STEC. Furthermore, diagnosis of non-O157 STEC is complex and currently requires a sequential approach [11,12] that entails screening for Shiga toxin or its encoding genes by non-cultural methods, followed by culture, colony identification and serotyping of the respective strain. Unfortunately, some countries lack recommendations for detecting non-O157 STEC and, even in those that have them, screening for Shiga toxin (genes) appears underutilized [12,13]. Adding further to the problem, culture isolation and serotyping of non-O157 STEC is performed only in a few specialized laboratories. Consequently, diagnosis of STEC including serotype - the basic microbiological information needed for surveillance - occurs infrequently and is time-consuming. This delays or even prevents pathogen-specific outbreak detection. Herein lies a particular problem: pathogenic *E. coli *continue to evolve [14,15] through inter-bacterial transfer of genetic elements, for example, via bacteriophages, transposons and plasmids, and new and emerging STEC clones will likely belong to the group of (underdiagnosed) non-O157 STEC.

The main reservoir for STEC is ruminants, particularly cattle, and most large STEC outbreaks, irrespective of serotype, have been caused by contaminated food (including drinking water) [16-18]. Timeliness of public health surveillance is the key to implementing effective control measures. In foodborne outbreaks, this translates into the necessity for 1) early detection, 2) timely identification of the suspected food vehicle and 3) removing it from the market, accompanied by targeted consumer advice. Minimizing secondary spread is an additional public health task to halt the outbreak, as affected persons themselves then have become a potential source of infection to others [19].

## Public health response to the STEC O104:H4 outbreak in Germany

**Figure 1:**
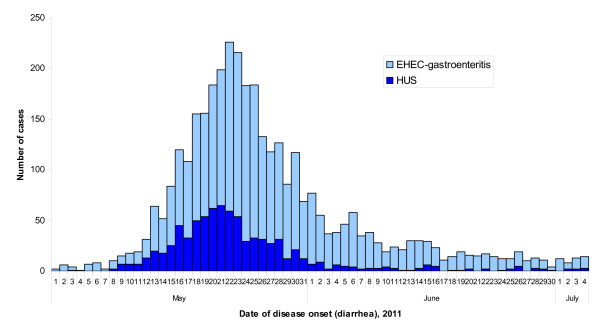
**Epidemic curve of a large outbreak of STEC O104:H4 infection in Germany, 2011**. lighter blue: reported cases of STEC gastroenteritis with available date of onset (n = 2,717) darker blue: reported cases of hemolytic uremic syndrome with available date of onset (n = 809). STEC, Shiga toxin-producing *Escherichia coli*

From early May through early July 2011, an international STEC outbreak occurred in Germany with the largest documented number of HUS cases in a single outbreak, predominantly occurring in adults [20]. In Germany, STEC infection and clinical symptoms compatible with diarrhea-associated HUS are notifiable. In the outbreak period, more than 3,800 cases, including 54 fatalities, were ascertained through Germany's national infectious disease reporting system. Of those, more than 800 developed HUS (Figure 1), severely straining nephrologic treatment capacities in the northern Germany outbreak area and beyond [21]. The causative agent was of a rare *E. coli *serotype, O104:H4, and has been classified as an enteroaggregative *E. coli *that had acquired Shiga toxin genes and other genetic elements [22-24]. Likely, lateral gene transfer has created a virulent hybrid clone with a blended virulence profile and an extended-spectrum β-lactamase phenotype [25,26].

### Outbreak detection

The outbreak was detected by a small cluster of three pediatric HUS patients immediately notified to a local health department - approximately two weeks after the outbreak started and before statistical algorithms on reporting data of laboratory-confirmed cases flagged an alert. This reiterates that small clusters can herald large outbreaks, thus exemplifying the role of alert clinicians in early outbreak detection.

### Identification of the suspected food

Evidence from epidemiologic studies and product 'tracing' investigations strongly implicated fenugreek sprouts as the cause of the STEC O104:H4 outbreak [27]. Identifying the suspect food was doubly complicated: First, by the unexpectedly long incubation period (median of eight days), which was for most cases outside the time-period for which food history was elicited in hypothesis-generating interviews. Second, sprouts were often used as garnish on meals or salads in restaurants or at catered festivities - therefore consumed sometimes only once during the exposure period, often unwittingly and without the memory aid of having purchased or prepared them. Consequently, initial hypothesis-generating interviews did not hint towards sprouts (only reported by 25% of cases), but foods that probably were often eaten in combination with them, such as leafy salads, cucumbers, or tomatoes [28]. A subsequent case-control study found a statistically significant association with sprouts. However, 75% of the cases denied their consumption, implicitly questioning the causal role of sprouts. Strong epidemiologic evidence for the outbreak vehicle was not obtained until an analytical investigation studied groups visiting a particular restaurant to which several cases could be attributed. Participants basically had to remember 'only' their meal - the ingredient list was supplied by the restaurant's chef. This study identified sprout consumption as the only significant risk for becoming ill - all cases had been served sprouts [27]. Further evidence was provided by food safety authorities. In sprout 'tracing' investigations, 41 dispersed infection sites (mainly restaurants) converged on a single sprout producing farm in Lower Saxony from which they had received sprout shipments [27]. Evidence from federal-level coordinated product tracing and epidemiological studies emerged in parallel leading to identification of sprouts as the most likely vehicle of infection, three weeks after the outbreak was detected (and five days after Lower Saxony's Minister of Agriculture publicly suspected sprouts based on preliminary results of sprout trace-back investigations).

### Outbreak control

On 10 June, a national public advisory, issued jointly by federal public health and food safety authorities, recommended that consumers should avoid sprout consumption. Simultaneously, implicated sprouts were taken off the market. Later, an international investigation concluded that a particular lot of fenugreek sprout seeds imported from Egypt in 2009 was the common link between the German outbreak and a related STEC O104:H4 cluster in south-west France [29,30]. Consumer advice was tailored accordingly. At the time of writing, the chain of events that led to contamination of the seeds with STEC O104:H4 remains unclear, but this information is pivotal for devising rational strategies to prevent similar events in the future [31]. Recommendations to prevent secondary spread in the household were based primarily on hygienic advice targeting affected persons or caregivers. Notwithstanding, secondary transmission, mainly in households, has been reported in noticeable frequency [32].

In this unprecedented outbreak of STEC O104:H4, statutory HUS surveillance facilitated outbreak detection and compensated for laboratory surveillance of STEC infection in Germany, which is based mainly on detection of Shiga toxins or their encoding genes and currently too often is terminated before pathogen isolation and characterization [33]. Once detected, public health response was intense and involved a series of epidemiologic investigations, supplemented by the tracing investigations of food safety authorities that provided strong epidemiologic evidence for sprouts as the vehicle in this outbreak. Even in hindsight, it is unlikely that this process could have been substantially accelerated because investigations that convincingly pointed to the outbreak vehicle could not be conducted before likely points of infection (of sufficient size for the ingredient-level study) were identified, which required thorough exposure assessment of cases at the local level and supra-regional collation and exchange of this information.

## Challenges ahead in the control of STEC outbreaks - the need for speed

### Early detection: improving completeness and timeliness of STEC diagnosis and subtyping

Countries vary in their surveillance approach towards STEC, partly due to the diagnostic challenges mentioned previously. As the evolution of virulent microbes continues, so does the development of diagnostic methods. For example, new screening tools allow the simultaneous assessment of virulence markers and HUS-relevant serogroups in one diagnostic step (for example, [34]). Furthermore, rapid genome sequencing techniques, already applied successfully during this outbreak [22-24], have recently found their way into the armamentarium of microbiologists, allowing for detailed strain characterization.

Integration of molecular subtyping information into public health surveillance (currently requiring culture isolation) is powerful. It greatly enhances sensitivity and timeliness of outbreak detection and focuses investigations by separating outbreak-related cases from geographically and temporally associated sporadic cases [35]. These striking advantages have transformed public health in countries that routinely make use of subtyping information, for example, the US [36]. Yet, growing budget restrictions, concerns about cost-effectiveness, and focussing on other public health priorities have hampered their implementation in many countries, including Germany. At any rate, strategies to improve timeliness of STEC diagnosis and typing need to be complemented by efforts to increase clinical suspicion of STEC infection in community-acquired diarrhea, particularly when bloody [37]. Considering the vagaries and underutilization of current STEC diagnostics, and the threat of emerging virulent *E. coli*, complimentary surveillance systems are warranted, such as syndromic surveillance of bloody diarrhea [38] or of diarrhea-associated HUS [12], almost exclusively caused by STEC. It is noteworthy that many large STEC outbreaks have initially been detected by small clusters of HUS [18,39,40], which is particularly true for the sorbitol-fermenting clone of O157:H-, where occurrence of diarrheal cases seems to be comparatively seldom [41]. Thus, the crucial role of alert physicians who secure microbiological diagnosis and timely inform public health authorities about clusters of illness cannot be overstated.

### Identifying suspect foods: Building on patients' recall is good, supplementing it is better

Epidemiologic identification of the suspected outbreak vehicle hinges on patients' food histories, but their incomplete recall often leads to inaccurate exposure characterization. In this context, timely identification and investigation of localized clusters of cases, even in geographically dispersed outbreaks, is pivotal for two reasons: first, clusters may provide information on place of infection, which can be used for product tracing investigations to identify (or rule out) the most likely outbreak vehicle. These kinds of investigations currently lack a standardized framework for their conduct. Second, clusters can provide valuable opportunities to supplement patients' memories [42], for example, in recipe-based studies [42]. Similarly, purchase information, for example, from grocery receipts [43], membership cards of store chains [44] or credit cards [45] as a surrogate for patients' food histories is increasingly used for hypothesis generation or testing. These epidemiologic tools can markedly increase the specificity of the outbreak investigation. As a corollary, food sampling strategies can be tailored accordingly, thereby enhancing the likelihood of obtaining microbiological evidence for the suspected food.

Traditional analytical epidemiologic investigations often employ case-control studies. They require selection of a valid control group, whose members are unaffected by the outbreak but are representative of outbreak patients with respect to food consumption prevalences. This selection process is frequently time-consuming and accompanied by logistical or legal difficulties, for example, accessing population registries. If estimates exist about the background consumption rate of specific foods (for example, through population surveys) or an educated guess about their range can be ventured, associations of food items with illness can be rapidly assessed by employing binomial probability theory before or even instead of using actual responses of controls [46,47]. Additionally or alternatively, making use of the exposure experience of patients with a similar disease, for instance outbreak-unrelated cases of the same serotype, merits further evaluation. Its usefulness would be another argument for conducting subtyping surveillance, which provides such 'control' patients.

### Controlling the outbreak: beyond food recall and warning - preventing secondary spread

Food recall and public warnings are standard prevention measures in the control of foodborne outbreaks. The key question is how much evidence is needed for a suspected food before these measures are warranted. Difficulties in the prevention of secondary transmission of STEC O157 to children, mainly occurring in the household, have led to far-reaching recommendations such as prompt separation of siblings [48] or even hospitalization of pediatric cases on clinical grounds [49]. This outbreak primarily affected adults who, compared to children, are less likely to transmit the pathogen to other persons [48,50]. The observation in Germany of adult-to-adult and adult-to-child household transmission even two months after the food vehicle had been removed from the market is notable. This calls for an in-depth analysis of intra-household transmission to improve recommendations for its prevention.

## Conclusions

STEC outbreaks, if large in size, are usually foodborne. Rapid identification of the contaminated food is essential for effective outbreak control. This requires a complex interplay of alert clinicians, microbiologists, and public health and food safety specialists. In each of these professions or disciplines, there is potential for improvement. Notwithstanding, one challenge will remain: controlling an ever-moving target - constantly evolving diarrheagenic *E. coli*.

## Competing interests

The authors declare that they have no competing interests.

## Authors' contributions

All authors participated in drafting and revising the manuscript, and they all read and approved the final manuscript.

## Pre-publication history

The pre-publication history for this paper can be accessed here:

http://www.biomedcentral.com/1741-7015/10/11/prepub
